# RhFGF21 Protects Epidermal Cells against UVB-Induced Apoptosis through Activating AMPK-Mediated Autophagy

**DOI:** 10.3390/ijms232012466

**Published:** 2022-10-18

**Authors:** Yeli Zhao, Jingjing Lin, Jiana Li, Canol Bwalya, Yuyun Xu, Yue Niu, Yujie Zhang, Junyi Wu, Yifan Xu, Jun Chen, Shasha Ye, Li Lin

**Affiliations:** 1 School of Pharmaceutical Science, Wenzhou Medical University, Wenzhou 325035, China; 2 Wenzhou TCM Hospital Affiliated to Zhejiang, Wenzhou 325000, China; 3 Research Units of Clinical Translation of Cell Growth Factors and Diseases Research, Chinese Academy of Medical Science, Wenzhou 325035, China

**Keywords:** UVB, rhFGF21, apoptosis, autophagy, AMPK

## Abstract

Ultraviolet irradiation, especially ultraviolet B (UVB) irradiation, increases the risks of various skin diseases, such as sunburn, photo-aging and cancer. However, few drugs are available to treat skin lesions. Therefore, the discovery of drugs to improve the health of irradiated skin is urgently needed. Fibroblast growth factor 21 (FGF21) is a metabolic factor that plays an important role in the protection and repair of various types of pathological damage. The effects of FGF21 on skin injury caused by UVB-irradiation were the focus of this study. We found that UVB irradiation promoted the expression of FGF21 protein in mouse epidermal cells, and exogenous recombinant human FGF21 (rhFGF21) protected mouse skin tissue against UVB-induced injury. RhFGF21 inhibited the inflammatory responses and epidermal cell apoptosis as well as promotion of autophagy in UVB-irradiated mice. Moreover, we found that rhFGF21 protected HaCaT cells against UVB-induced apoptosis, and the protective effect was enhanced by treatment with an autophagy activator (rapamycin) but was inhibited by treatment with an autophagy inhibitor (3-methyladenine, 3MA). AMP-activated protein kinase (AMPK), as a cellular energy sensor, regulates autophagy. RhFGF21 increased the expression of p-AMPK protein in epidermal cells irradiated with UVB in vivo and in vitro. Moreover, rhFGF21 increased autophagy levels and the viability were diminished by treatment with an AMPK inhibitor (compound C). RhFGF21 protects epidermal cells against UVB-induced apoptosis by inducing AMPK-mediated autophagy.

## 1. Introduction

The skin is the first line of defense against harmful biological, physical and environmental pollutants, including ultraviolet (UV) radiation [1]. UV radiation is classified by wavelength as UVA (315–400 nm), UVB (280–315 nm) and UVC (100–280 nm) [2]. UV irradiation exposure, especially UVB exposure, increases the risks of various skin diseases, such as cancer, photo-aging, sunburn and pigmentation [3,4]. UVB influences the functions of epidermal keratinocytes and dermal fibroblasts by triggering DNA breakage, inflammation, oxidative stress and apoptosis [5,6]. Currently, the most powerful protection against UVB irradiation is sunscreen. However, the active ingredients in the sunscreen only prevent the effects of direct solar sunshine [2,7]. To fully protect skin from UVB-induced damage, the discovery of drugs to improve the health of irradiated skin is needed.

Fibroblast growth factor 21 (FGF21) is a member of the fibroblast growth factor (FGF) family that is abundantly expressed in liver and skin tissues [8,9]. FGF21 plays an important role in the regulation of various physiological and pathological processes, such as embryonic development, cell migration, proliferation and differentiation [10]. Currently, studies on FGF21 have mainly focused on tissue repair and functional protection [11,12,13]. Our previous study revealed that recombinant human FGF21 (rhFGF21) promoted wound healing in diabetic mice [14]. It has been reported that FGF21 protects the blood–brain barrier from traumatic brain injury [15]. However, whether FGF21 protects skin tissues against UVB irradiation effects is unclear. Therefore, the effects of FGF21 on UVB-injured skin were the focus of this study.

Autophagy, a highly conserved self-digestion pathway, recycles the damaged components to produce energy and constituents or induces cell death [16,17]. Autophagy is triggered by various cellular stresses, such as starvation, hypoxia and DNA damage [17]. An in vitro study indicated that UVB exposure reduced autophagy in human epidermoid carcinoma A431 cells [2]. However, the role of autophagy in cells exposed to UVB radiation is unclear. AMP-activated protein kinase (AMPK), as a cellular energy sensor, is involved in cellular processes including autophagy and apoptosis [18,19]. It has been reported that silibinin increases autophagy in murine 3T3-L1 preadipocytes by activating the AMPK signaling pathway [20]. FGF21 promotes ischemic angiogenesis and endothelial progenitor cell function under diabetic conditions by activating the AMPK/NAD pathway [21]. FGF21 augments autophagy in random-pattern skin flaps via AMPK signaling pathways and improves tissue survival [22]. Therefore, we hypothesized that AMPK-mediated autophagy plays a role in rhFGF21-mediated protection of skin injury caused by UVB irradiation.

Considering the aforementioned data, in the present study, we aimed to investigate the protective effects and the potential mechanisms of rhFGF21 on UVB-induced epidermal injury in vitro and in vivo. We found that rhFGF21 protected epidermal cells against UVB-induced apoptosis by activating AMPK-mediated autophagy.

## 2. Results

### 2.1. Treatment with RhFGF21 Protected Mouse Skin Tissue against UVB-Induced Injury

**Figure 1:**
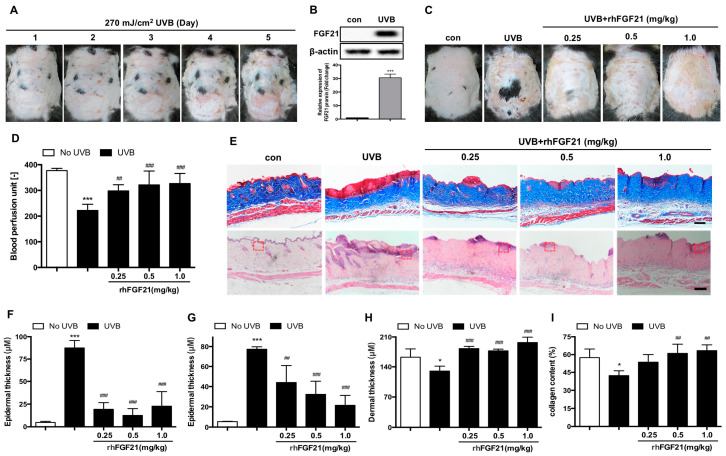
Treatment with rhFGF21 protects skin tissue against UVB-induced injury in mice. (**A**) Representative images of mouse dorsal skin irradiated with different UVB doses. (**B**) The level of FGF21 protein was detected by Western blot analysis. Quantitative analysis of the intensity of FGF21. (**C**) Representative images of rhFGF21-protected skin tissue after UVB irradiation in mice. (**D**) Blood perfusion in the damaged skin area was measured by a laser Doppler flowmeter. (n = 6). (**E**) Masson staining and HE staining. Local enlarged pictures (red dash box) were shown in Figure 2A. Scale bar = 100 μm. (**F**,**G**) Quantification of the epidermal thickness in Masson staining and HE staining. (n = 4). (**H**) Quantification of the dermal thickness. (n = 4). (**I**) Quantification of collagen content in skin tissues. (n = 4). The data are presented as means ± SD. * *p* < 0.05, *** *p* < 0.001 versus mice without UVB irradiation exposure; ^##^ *p* < 0.01, ^###^ *p* < 0.001 versus UVB-irradiated mice.

Mouse skin was exposed to 270 mJ/cm^2^ UVB (approximately 1.5 h) for 5 consecutive days. After irradiation with UVB for a different number of days, the mouse skin tissues were damaged, and the degrees of skin injury were more severe at higher UVB exposure (Figure 1A). In addition, skin injury was more obvious when the skin was irradiated with UVB for 5 consecutive days. Therefore, the method used in the present study was based on these preliminary experimental results. Next, we detected whether UVB irradiation affected the expression of FGF21 protein in skin tissues. The results showed that UVB irradiation induced FGF21 expression in mice, compared to the control mice (Figure 1B). The results suggested FGF21 plays an important role in UVB-induced skin injury.

It is well known that FGF21 plays a positive role in tissue repair and functional protection [11,12,13]. Therefore, we examined the protective effect of rhFGF21 on UVB-induced skin injury in mice. In contrast to the skin of untreated mice, the skin of mice subjected to UVB irradiation was injured. However, rhFGF21 significantly improved skin injury in UVB-irradiated mice (Figure 1C). UVB-induced skin injury is associated with blood vessel damage; therefore, we detected blood flow in the skin through Laser Doppler flowmetry to evaluate the degree of skin injury. The Doppler-measured skin blood flow revealed that UVB-induced blood perfusion inhibition was attenuated by rhFGF21 treatment (Figure 1D). Masson staining and HE staining showed that rhFGF21 inhibited epidermal thickness in UVB-irradiated mice. Moreover, Masson staining revealed that rhFGF21 increased dermal thickness and collagen content in UVB-irradiated mice (Figure 1E–I). These results suggested that rhFGF21 improved UVB-induced skin injury in mice.

### 2.2. RhFGF21 Inhibited the Inflammatory Cell Infiltration in UVB-Irradiated Mouse Skin

**Figure 2:**
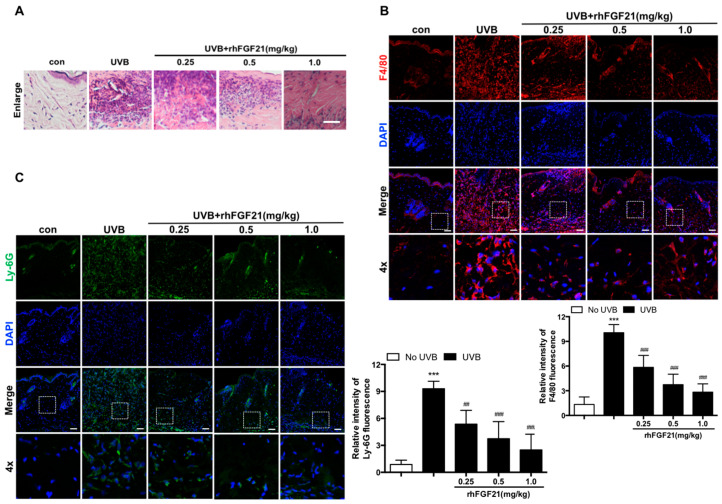
RhFGF21 inhibited inflammatory cell infiltration in UVB-irradiated mouse skin. (**A**) HE staining (n = 4). Scale bar = 25 μm. Local enlarged picture in HE staining (Figure 1E). (**B**,**C**) Immunofluorescence confocal microscopy images of F4/80 and Ly-6G in UVB-irradiated mouse skin tissues. Scale bar = 100 μm. The Data are presented as means ± SD. *** *p* < 0.001 versus mice without UVB-irradiation exposure; ^##^ *p* < 0.01, ^###^ *p* < 0.001 versus UVB-irradiated mice. The data are presented as means ± SD. *** *p* < 0.001 versus mice without UVB irradiation exposure; ^##^ *p* < 0.01, ^###^ *p* < 0.001 versus UVB-irradiated mice.

In the present study, the pathological changes in skin tissues were observed under a light microscope after HE staining. Based on HE staining (Figure 1E) and its local enlarged picture (Figure 2A), we found that UVB irradiation induced considerable inflammatory cell infiltration in skin tissues. In contrast, inflammatory cell infiltration was inhibited by rhFGF21 administration (Figure 2A). Neutrophils and macrophages are the main innate immune cells in the body. Once activated, they migrate into lesion tissues to mediate inflammatory responses. Thus, we investigated the effect of rhFGF21 on the infiltration of neutrophils and macrophages by Ly6G and F4/80 immunofluorescence staining, respectively. We found that rhFGF21 significantly inhibited the infiltration of neutrophils and macrophages in UVB-irradiated mice (Figure 2B,C). These results revealed that rhFGF21 inhibited UVB irradiation-induced inflammatory cell infiltration in skin tissues.

### 2.3. RhFGF21 Inhibited the Inflammatory Response and Epidermal Cell Apoptosis in UVB-Irradiated Mouse Skin

**Figure 3:**
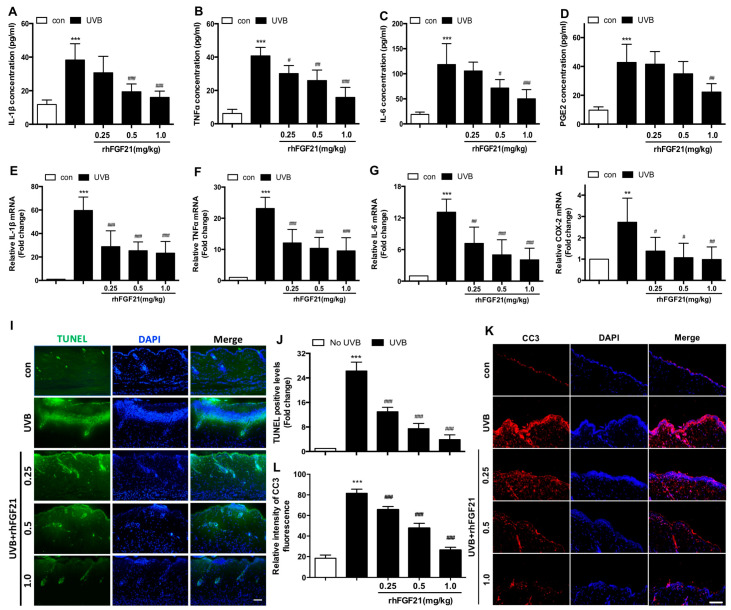
RhFGF21 inhibited inflammatory response and epidermal cell apoptosis in UVB-irradi ated skin in mice. (**A**–**D**) Levels of IL-1β, TNFα, IL-6 and PGE2 in mouse serum were measured by ELISA kits. (n = 6). (**E**–**H**) The mRNA levels of IL-1β, TNFα, IL-6 and COX-2 in skin tissues were measured by RT-PCR. (n = 6). (**I**,**J**) Cell apoptosis in damaged skin tissues was evaluated by TUNEL staining. Scale bar = 50 μm. The apoptosis-positive cell levels were quantified. (n = 3). (**J**,**K**) The expression of cleaved caspase-3 (CC3) was analyzed and quantified by confocal microscopy and Image J, respectively. Red fluorescence represents the expression of CC3 protein, blue fluorescence represents nucleus DAPI. Scale bar = 100 μm. Data are presented as means ± SD. ** *p* < 0.01, *** *p* < 0.001 versus mice without UVB-irradiation exposure; ^#^ *p* < 0.05, ^##^ *p* < 0.01, ^###^ *p* < 0.001 versus UVB-irradiated mice.

Immune cells-mediated inflammatory response has been characterized as an essential factor in regulating the progression of skin injury [1]. Therefore, the levels of pro-inflammatory mediators in the serum and skin tissues were measured by ELISA and RT-PCR assays, respectively. UVB irradiation significantly promoted the secretion of IL-1β, TNFα, IL-6 and PGE2 in mouse serum, and the levels of all these pro-inflammatory mediators were suppressed by rhFGF21 treatment (Figure 3A–D). Moreover, the increased levels of IL-1β, TNFα, IL-6 and COX-2 mRNA stimulated by UVB-irradiated skin tissues were all suppressed by rhFGF21 treatment (Figure 3E–H).

UVB irradiation is a major cause of skin damage through the induction of skin cell apoptosis [23]. TUNEL staining is extensively used in apoptosis research. Therefore, we sought to determine whether rhFGF21 protects skin injury from cell apoptosis by using TUNEL staining. UVB irradiation induced epidermal cell apoptosis, and this effect was inhibited by rhFGF21 administration (Figure 3I,J). Moreover, we detected the expression of cleaved caspase-3 protein by immunofluorescence staining. RhFGF21 inhibited the expression of cleaved caspase-3 protein in UVB-irradiated mice (Figure 3K,L). These results indicated that rhFGF21 inhibited the inflammatory response and epidermal cell apoptosis in UVB-treated mice. Further study was directed to the protective mechanism of rhFGF21 on epidermal cells in UVB-irradiated mice. Therefore, HaCaT cells, as human immortalized keratinocytes, were selected for subsequent in vitro mechanistic studies.

### 2.4. Treatment with RhFGF21 Protected HaCaT Cells from UVB-Induced Apoptosis

**Figure 4:**
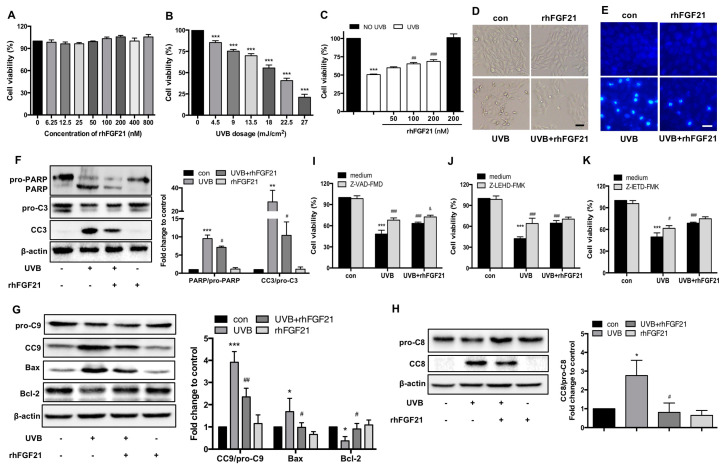
Treatment with rhFGF21 protects HaCaT cells from UVB-induced apoptosis. (**A**) The effect of rhFGF21 on HaCaT cell viability. Cell viability was assessed by MTT assay. (**B**) The effect of UVB on the viability of HaCaT cells. Cell viability was assessed by MTT assay. (**C**) The effect of rhFGF21 on the viability of UVB-irradiated HaCaT cells. Cell viability was assessed by MTT assay. (**D**) Morphological images observed by phase contrast microscopy. Scale bar = 50 μm. (**E**) Fluorescence microscopic images of cells stained with Hoechst 33258. Scale bar = 25 μm. (**F**) Western blot analysis of PARP, pro-caspase-3 (pro-C3) and active caspase-3 (CC3). Quantitative analysis of the intensity of PARP, pro-C3 and CC3. (**G**,**H**) The protein levels of pro-caspase-9, active caspase-9, Bax, Bcl-2, pro-caspase-8 (pro-C8) and active caspase-8 (CC8) were detected and quantified by Western blot and ImageJ, respectively. (**I**–**K**) Viability of cells treated with Z-VAD, Z-LEHD and Z-IETD, respectively. Cell viability was evaluated by MTT assay. Data are the means ± SD of three independent experiments, * *p* < 0.05, ** *p* < 0.01, *** *p* < 0.001 versus HaCaT cells without UVB radiation exposure; ^#^ *p* < 0.05, ^##^ *p* < 0.01, ^###^ *p* < 0.001 versus UVB-irradiated HaCaT cells; ^&^ *p* < 0.05 versus rhFGF21-treated HaCaT cells injured by UVB irradiation.

First, we examined the effects of rhFGF21 on HaCaT cell viability. We found that rhFGF21 at concentrations ranging from 6.25 to 800 nM did not influence the viability of HaCaT cells (Figure 4A). Therefore, rhFGF21 within this safe concentration range was selected for study in UVB-irradiated HaCaT cells. Next, the dose-dependent cytotoxic effects of UVB in HaCaT cells were observed. Cell viability decreased with increasing UVB dosages, and the growth inhibitory rate of UVB on HaCaT cells was approximately 55% at a dose of 18 mJ/cm^2^ (Figure 4B). Therefore, UVB irradiation at a dose of 18 mJ/cm^2^ was selected for further study. The results of the MTT assay revealed that rhFGF21 treatment enhanced the viability of UVB-irradiated cells in a dose-dependent manner (Figure 4C). Moreover, 100 nM and 200 nM rhFGF21 significantly rescued HaCaT cells from UVB-induced damage, and there were no obvious differences in the protective effect of rhFGF21 at concentrations from 100 nM to 200 nM (Figure 4C). Therefore, rhFGF21 at a concentration of 100 nM was selected for further study in UVB-irradiated HaCaT cells.

The features of cellular apoptosis after UVB irradiation, such as cell shrinkage and the formation of apoptotic bodies, were observed under a microscope, and these changes were reversed by rhFGF21 treatment (Figure 4D). Hoechst 33258 is a stain used to detect DNA fragmentation resulting from apoptotic signaling cascades. UVB irradiation significantly increased the number of Hoechst-positive cells, and this increase was reversed by rhFGF21 treatment (Figure 4E). Western blot analysis showed that UVB irradiation promoted the protein expression of activated caspase-3 (CC3) and PARP. However, this promotion was inhibited by rhFGF21 treatment (Figure 4F). Cell apoptosis is associated with nonmitochondrial- and mitochondrial-mediated signaling pathways. To further examine the protective mechanism of rhFGF21, we detected the expression levels of mitochondrial and nonmitochondrial apoptosis-associated proteins by Western blotting. RhFGF21 inhibited the expression of Bax, cleaved caspase-9 (CC9) and cleaved caspase-8 (CC8) induced by UVB irradiation, and reversed the expression of Bcl-2 protein that had been inhibited by UVB irradiation (Figure 4G,H). In addition, the viability of UVB-irradiated cells was enhanced through the inhibition of caspase-3, caspase-9 and caspase-8. Moreover, the inhibition of caspase-3 further increased the viability of rhFGF21-treated cells exposed to UVB irradiation (Figure 4I–K). Therefore, these results revealed that treatment with rhFGF21 protected HaCaT cells against UVB-induced apoptosis.

### 2.5. RhFGF21 Rescued UVB-Irradiated HaCaT Cells by Inducing Autophagy

**Figure 5:**
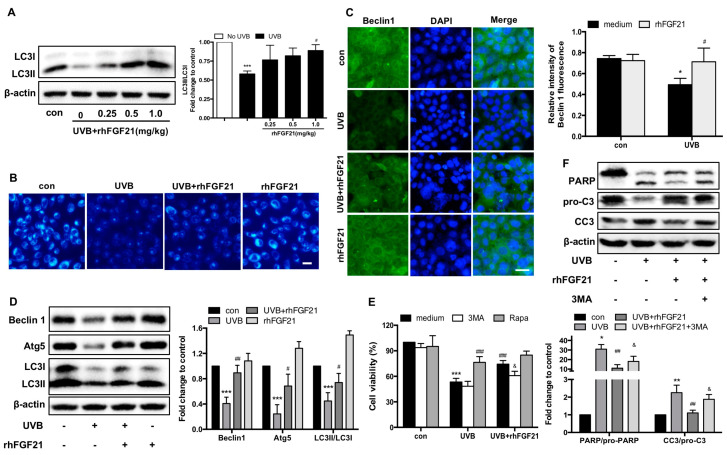
RhFGF21 rescues UVB-irradiated HaCaT cells by inducing autophagy. (**A**) The expression of LC3 protein was detected by Western blot analysis, and quantitative analysis of the intensity of LC3 II/LC3 I. (**B**) Cells were stained with MDC solution. Morphological changes were observed by fluorescence microscopy. Blue fluorescence represents autophagy levels. Scale bar = 25 μm. (**C**) Immunofluorescence confocal microscopy images of Beclin 1 in HaCaT cells. Green fluorescence represents the expression of Beclin 1 protein, blue fluorescence represents nucleus DAPI. Scale bar = 25 μm. Quantitative analysis of the intensity of Beclin 1. (**D**) The protein levels of Beclin 1, Atg5 and LC3 were detected by Western blot analysis. (**E**) Cells treated with or without an autophagy activator (rapamycin, Rapa, 20 μM) or autophagy inhibitor (3-methyladenine, 3MA, 1 mM). Cell viability was determined by MTT assay. (**F**) Western blot analysis of PARP, caspase-3 and active caspase-3 (CC3) levels. Quantitative analysis of PARP and CC3/pro-C3 levels. Data are means ± SD of three independent experiments, * *p* < 0.05, ** *p* < 0.01, *** *p* < 0.001 versus epidermal cells or HaCaT cells not exposed to UVB radiation; ^#^ *p* < 0.05, ^##^ *p* < 0.01, ^###^ *p* < 0.001 versus UVB-irradiated epidermal cells or HaCaT cells; ^&^ *p* < 0.05 versus rhFGF21-treated HaCaT cells injured by UVB irradiation.

Autophagy plays an essential role in cellular responses to stresses, such as starvation, hypoxia and DNA damage [17]. Hence, we sought to investigate whether there was a relationship between autophagy induction and UVB-induced cell apoptosis. First, the expression of LC3 protein in mouse epidermal cells was detected by Western blotting. RhFGF21 treatment increased LC3-II expression in UVB-irradiated mouse epidermal cells (Figure 5A). Next, autophagy levels in HaCaT cells were examined by MDC staining. We found that the percentage of MDC-positive cells was decreased in UVB-irradiated HaCaT cells, and this decrease was reversed by rhFGF21 treatment (Figure 5B). Immunofluorescence staining demonstrated that rhFGF21 increased the fluorescence intensity of Beclin 1 in UVB-irradiated HaCaT cells (Figure 5C). To further confirm this phenomenon, we examined the expression levels of autophagy-associated proteins by Western blotting. The protein expressions of Beclin 1, Atg5 and LC3-II were inhibited in UVB-irradiated HaCaT cells, and this inhibition was reversed by rhFGF21 treatment (Figure 5D). These results suggested that rhFGF21 promoted autophagy in UVB-irradiated HaCaT cells.

It has been reported that autophagy induced by silibinin protects human epidermoid carcinoma A431 cells from UVB-induced apoptosis [2] Therefore, we hypothesized that rhFGF21 rescues UVB-irradiated HaCaT cells by inducing autophagy. To verify our hypothesis, we added an autophagy inhibitor (3-methyladenine, 3MA) or autophagy activator (rapamycin) to the cell culture. We found that 3MA inhibited the viability enhancement effect of rhFGF21 in UVB-irradiated HaCaT cells, while rapamycin increased the viability enhancement effect of rhFGF21 in UVB-irradiated HaCaT cells (Figure 5E). Consistent with the cell viability results, rhFGF21 inhibited the expression of active PARP and cleaved caspase-3 protein in UVB-irradiated HaCaT cells, whereas 3MA reversed their expression (Figure 5F). These results revealed that rhFGF21-enhanced autophagy might play a positive role in cell apoptosis induced by UVB irradiation.

### 2.6. RhFGF21 Protected HaCaT Cells from UVB-Induced Apoptosis by Activating AMPK-Mediated Autophagy

**Figure 6:**
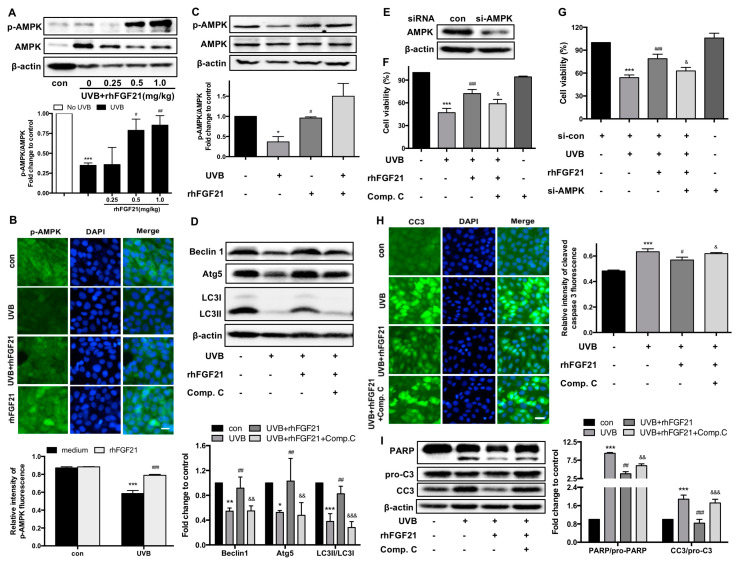
RhFGF21 protects HaCaT cells from UVB-induced apoptosis by activating AMPK-mediated autophagy. (**A**) The expression of p-AMPK and AMPK proteins in epidermal cells were detected by Western blot analysis, and quantitative analysis of the intensity of p-AMPK/AMPK. (**B**) Immunofluorescence confocal microscopy images of p-AMPK in HaCaT cells. Green fluorescence represents the expression of p-AMPK protein, blue fluorescence represents nucleus DAPI. Scale bar = 25 μm. Quantitative analysis of the intensity of p-AMPK. Scale bar = 25 μm. Quantitative analysis of the intensity of p-AMPK. (**C**) The expression of p-AMPK and AMPK proteins were detected by Western blot analysis, and quantitative analysis of the intensity of p-AMPK/AMPK. (**D**) Cells treated with AMPK inhibitor (Compound C, Comp. C, 1 mM). The protein levels of Beclin 1, Atg5 and LC3 were determined by Western blot and quantified by Western blot and ImageJ, respectively. (**E**) AMPK expression was silenced by transfection with siRNA. (**F**,**G**) Effect of inhibiting or silencing AMPK on cell viability. Comp. C, 1 mM; si-AMPK, 20 nM. (**H**) Cellular expression of active caspase-3 was analyzed by confocal microscopy. Green fluorescence represents the expression of active caspase-3 (CC3) protein, blue fluorescence represents nucleus DAPI. Scale bar = 25 μm. (**I**) The protein levels of PARP, pro-caspase 3 (pro-C3) and active caspase 3 (CC3) were detected by Western blot and quantified by Western blot and ImageJ, respectively. Data are means ± SD of three independent experiments, * *p* < 0.05, ** *p* < 0.01, *** *p* < 0.001 versus epidermal cells or HaCaT cells not exposed to UVB radiation; ^#^ *p* < 0.05, ^##^ *p* < 0.01, ^###^ *p* < 0.001 versus UVB-irradiated epidermal cells or HaCaT cells; ^&^ *p* < 0.05, ^&&^ *p* < 0.01, ^&&&^ *p* < 0.001 versus rhFGF21-treated HaCaT cells injured by UVB irradiation.

AMPK, as a cellular energy sensor, regulates autophagy [19]. Hence, we focused on the relationship between autophagy and AMPK activity. RhFGF21 increased p-AMPK expression in UVB-irradiated epidermal cells (Figure 6A). Moreover, rhFGF21 increased the fluorescence intensity and protein level of p-AMPK in UVB-irradiated HaCaT cells (Figure 6B,C). To confirm the role of AMPK in rhFGF21-induced autophagy, HaCaT cells were pretreated with the AMPK inhibitor compound C. The results showed that compound C treatment reduced the rhFGF21 restoration of autophagy-associated proteins (Beclin 1, Atg5 and LC3-II) in UVB-irradiated HaCaT cells (Figure 6D). These results suggested that rhFGF21 enhanced autophagy in UVB-treated HaCaT cells by activating AMPK.

To confirm the role of AMPK-mediated autophagy in rhFGF21-mediated protection against apoptosis, we added compound C or si-AMPK (Figure 6E) to the cell culture. We found that the inhibition of AMPK by compound C or si-AMPK treatment decreased the rhFGF21-restored cell viability of UVB-irradiated HaCaT cells (Figure 6F,G). Consistent with the results of cell viability, the fluorescence intensity of cleaved caspase-3 and the expression of apoptosis-associated proteins (cleaved caspase-3 and PARP) were all inhibited by compound C (Figure 6G,I). These results revealed that rhFGF21 protects cells against UVB-induced apoptosis by activating AMPK-mediated autophagy.

## 3. Discussion

Skin, as the largest organ in the human body, is the primary barrier against external injury [24]. UVB is a major environmental factor in skin damage and can lead to several pathologic changes, such as sunburn, erythema, oedema and skin cancer [25]. Currently, sunscreen can protect skin tissue from damage caused by UVB irradiation. However, sunscreen does not repair skin tissue damaged by exposure to UVB radiation [2]. Moreover, few drugs are available to treat skin lesions [26,27]. Fibroblast growth factor 1 (FGF1) and fibroblast growth factor 2 (FGF2) are common drugs used to treat skin lesions [28,29]. However, FGF1 and FGF2 increase the risk of tumorigenesis and scarring because of their mitogenic activity [30,31]. Therefore, the discovery of safe and effective drugs is urgently needed. FGF21, as a metabolic factor, is the only member of the FGF family with no mitogenic activity and thus can be safely used in clinical applications [14]. FGF21 protects cells from acute injury in several kinds of cells, such as endothelial cells, cardiomyocytes, and islet β cells [32,33]. However, whether FGF21 can protect skin from UVB damage is unknown. Therefore, in the present study, we aimed to study the effects of rhFGF21 on UVB-irradiated skin injury in mice. We found that rhFGF21 can protect skin tissue against UVB-induced injury in mice. Moreover, rhFGF21 improved the epidermal thickness and collagen content in UVB-irradiated mice. These results suggested that rhFGF21 is a potential drug for treating UVB-induced skin damage. In addition, FGF1 and FGF2 are commonly used to treat skin lesions through local application on the skin. This suggested that FGF21 protected skin injury from UVB irradiation through local application. Thus, we will conduct a series of experiments in the future to investigate the protective effects of local rhFGF21 application on UVB-irradiated mice. However, FGF1, FGF2 and FGF21 have some common characteristics, such as short-half life and poor stability. FGF21 requires repeated administration to achieve a therapeutic effect. Therefore, sustained-release materials for a combination with rhFGF21 should be identified to overcome the shortcoming of the need for repeated administration.

UVB irradiation induces skin injury through several deleterious signaling pathways, including the induction of apoptosis, inflammation, reactive oxygen species (ROS) and collagen degradation [23,34]. UVB irradiation induces acute inflammatory responses in skin by promoting the release of pro-inflammatory mediators such as TNFα, IL-1β, IL-6 and PGE2 [35]. Therefore, the repression of apoptosis and inflammation are critical for treating skin damage caused by UVB irradiation. FGF21 exerts multiple biological functions through its anti-apoptosis and anti-inflammation effects [33]. FGF21 protects the heart from myocardial ischemia and ischemia/reperfusion (I/R) injury by inhibiting apoptosis and inflammation [36]. Moreover, it has been reported that FGF21 protects dopaminergic neurons in Parkinson′s disease models by repressing neuroinflammation [32]. Considering this previous evidence, we investigated the effect of rhFGF21 on apoptosis and inflammation in UVB-irradiated mice. We found that rhFGF21 inhibited epidermal cell apoptosis and the production of pro-inflammatory mediators in UVB-irradiated mice. These results suggested that anti-apoptosis and anti-inflammatory mediators are involved in skin tissue protection in rhFGF21-treated mice under UVB irradiation.

FGF21 mediates the protective effect of fenofibrate against acetaminophen-induced hepatotoxicity by activating autophagy in mice [37]. Autophagy plays a key role in degrading aggregated or damaged proteins and organelles [38]. Autophagy is regulated by the AMPK signaling pathway [38,39]. AMPK, a serine–threonine kinase, is a well-studied metabolic energy sensor and regulator [38]. It has been reported that the AMPK pathway is inhibited in UVB-irradiated mouse skin, and AMPK deletion inhibited DNA repair [40]. The predominant DNA photoproducts caused by UVB radiation are pyrimidine (6-4) pyrimidone dimers (6-4PP) and cyclobutane pyrimidine dimers (CPDs). CPDs are also a major source of DNA breaks that cause genomic instability [40]. This suggested that AMPK is required for the efficient repair of UVB-induced DNA damage, linking energy metabolism with genomic stability. FGF21 augments autophagy in random-pattern skin flaps via the AMPK signaling pathway [22]. This evidence suggests that rhFGF21 protects epidermal injury and might be associated with AMPK-regulated autophagy. However, the role of rhFGF21 in AMPK-mediated autophagy in UVB-irradiated epidermal cells remains unclear. Therefore, we first investigated the effect of rhFGF21 on AMPK-mediated autophagy in UVB-irradiated HaCaT cells. We found that rhFGF21 promoted autophagy in UVB-irradiated HaCaT cells by activating AMPK signaling. FGF21 protects the heart from myocardial ischemia and I/R injury by activating the AMPK signaling pathway, and the underlying mechanism is associated with anti-apoptotic effects [41]. As we know, autophagy can regulate apoptosis [2,42,43]. Therefore, we sought to investigate whether AMPK-mediated autophagy is involved in the inhibitory effect of rhFGF21 on apoptosis. We found that rhFGF21 inhibited apoptosis by activating the AMPK-mediated autophagy signaling pathway. These findings suggested that rhFGF21 inhibited apoptosis in UVB-irradiated epidermal cells by activating the AMPK-mediated autophagy signaling pathway.

In conclusion, this study suggested a protective effect of rhFGF21 on skin damage induced by UVB irradiation, and we found that rhFGF21 protected HaCaT cells against UVB-irradiated injury by activating the AMPK-mediated autophagy signaling pathway, and the mechanism was determined to be at least partially associated with anti-apoptotic effects.

## 4. Materials and Methods

### 4.1. Reagents

RhFGF21 was extracted and purified from *Escherichia coli* (*E. coli*) as previously described [44]. Methylthiazolyldiphenyl-tetrazolium bromide (MTT), monodansylcadaverine (MDC) and Hoechst 33258 were obtained from Sigma Chemical (St. Louis, MO, USA). 3-Methyladenine (3MA), rapamycin, Z-VAD, Z-LEHD and Z-IETD were obtained from Selleckchem (Houston, TX, USA). Enzyme-linked immunosorbent assay (ELISA) diagnostic kits for mouse IL-1β, TNFα and PGE2 were purchased from Dakewe Biotech (Shenzhen, Guangdong, China). Short interfering RNA (siRNA) targeting human AMPK (si-AMPK) and negative control siRNA (si-con) were purchased from GenePharma (Suzhou, Jiangsu, China). Primary antibodies against cleaved caspase-3 (No. 9661s) and p-AMPKα (No. 2535s) were purchased from Cell Signaling Technology (Danvers, MA, USA). Primary antibodies against AMPK (No. 10929-2-AP), PARP (No. 66520-1-Ig), caspase-8 (No. 13423-1-AP), caspase-9 (No. 66169-1-Ig), caspase-3 (No. 19677-1-AP), Bax (No. 50599-2-Ig), Bcl-2 (No. 26593-1-AP), Beclin 1 (No. 66665-1-Ig), Atg5 (No. 66744-1-Ig) and LC3 (No. 18725-1-AP) were purchased from Proteintech Biotechnology (Wuhan, Hubei, China). Horseradish peroxidase-conjugated goat anti-rabbit IgG (No. ab6721), horseradish peroxidase-conjugated rabbit anti-mouse IgG (No. ab6728), primary antibodies against β-actin (No. ab8227) and an AMPK inhibitor (compound C) were purchased from Abcam (Cambridge, MA, USA). Electrochemiluminescence (ECL) reagent was purchased from Thermo Scientific (Rockford, IL, USA).

### 4.2. Experimental Protocol In Vivo

C57BL/6 mice (6–8 weeks old, weighing 18–22 g) were purchased from the Animal Center of the Chinese Academy of Sciences (Beijing, China). All animal experiments were performed following approval from the Animal Research Ethics Committee of Wenzhou Medical University and were in accordance with the National Institutes of Health guidelines concerning the care and use of laboratory animals. Mice were raised at a constant temperature with 12 h light/dark cycles and allowed free access to food and water.

The animals were randomly divided into five groups. The mice were anesthetized by isoflurane, the hair on the dorsum area was shaved with an electric clipper and depilatory creams were used to remove the residual hair. Next, the mice were exposed to UVB lamps (220 V, 20 W) (Beijing Lighting Research Institute, Beijing, China) equipped with a controller to modulate the UVB dosage. Mouse skin was exposed to 270 mJ/cm^2^ UVB irradiation (approximately 1.5 h) once a day for 5 consecutive days. RhFGF21 was administered with intraperitoneal injection in doses of 0.25, 0.5 and 1.0 mg/kg following daily UVB irradiation. The following treatment groups were generated: normal mice (con); UVB-irradiated mice (UVB); and rhFGF21-treated mice by intraperitoneal injection after UVB irradiation (UVB + rhFGF21). RhFGF21 was administrated at 0.25, 0.5 and 1.0 mg/kg. On day 6, all the mice were sacrificed after body weights and blood glucose levels were measured. Blood and skin tissue samples were harvested for further examination. Serum was obtained from blood by centrifugation at 3500 rpm for 10 min at 4 °C and then stored at −20 °C.

### 4.3. Laser Doppler Flowmetry

Mice were anesthetized with isoflurane and fixed in a stereotactic frame. On day 6, blood perfusion in each mouse was measured in four random fields of the injured regions with a laser Doppler fiber (Oxford Optronix, Oxford, UK) at 6 day.

### 4.4. Histopathological Analysis

Skin tissue specimens fixed with 4% paraformaldehyde were embedded in paraffin and sliced into 5-μm-thick sections. The skin tissues were stained with hematoxylin and eosin (HE) (Beyotime Institute of Biotechnology, Nanjing, China) for epidermal thickness evaluation and with Masson′s trichrome (Solarbio, Beijing, China) for epidermal thickness, dermal thickness and dermal collagen formation assessment. Quantification was carried out for percentage changes of collagen content in skin tissue. Images were captured using a light microscope (Nikon, Tokyo, Japan) and analyzed with ImageJ (NIH) software (National Institutes of Health, Bethesda, MD, USA).

### 4.5. TUNEL Assay

To analyze the apoptosis rate by terminal deoxynucleotidyl transferase dUTP nick end labeling (TUNEL) assays, skin tissues were permeabilized with 33% acetic acid in ethanol at −20 °C for 5 min and then labeled with an apoptosis detection kit (Beyotime Institute of Biotechnology, Nanjing, China) at 37 °C for 1 h. Skin tissues were washed three times with phosphate-buffered saline (PBS) and stained with 4′,6-diamidino-2-phenylindole (DAPI) for 10 min. DAPI was included as a nuclear marker. Images were photographed with a fluorescence microscope (Olympus, Tokyo, Japan) and quantitatively analyzed with ImageJ software (National Institutes of Health, Bethesda, MD, USA).

### 4.6. Measurement of IL-1β, TNFα, IL-6 and PGE2 Levels

The levels of IL-1β, TNFα, IL-6 and PGE2 were determined with enzyme-linked immunosorbent assay (ELISA) detection kits according to the manufacturer′s instructions (Dakewe Biotech, Shenzhen, Guangzhou, China).

### 4.7. The Epidermal Cell Extraction

Mice were sacrificed after anesthesia, and skin tissue was collected for analysis. The samples were cut into pieces and then digested with 0.1% collagenase (Biosharp, Shanghai, China) at 37 °C for 1 h, followed by centrifugation at 12,000 rpm at 4 °C for 10 min. The precipitate was lysed on ice with protein extraction reagents (Beyotime Institute of Biotechnology, Shanghai, China) for 1.5 h and then centrifuged at 12,000 rpm at 4 °C for 10 min. The supernatant was then removed and stored at −20 °C. The supernatant samples were separated and detected by Western blotting.

### 4.8. Cell Culture

Human immortalized keratinocyte (HaCaT) cells were purchased from BIOBW (Beijing, China). The cells were cultured in modified Eagle’s medium (MEM) supplemented with 15% fetal bovine serum (FBS) (Gibco, Grand Island, NE, USA) and 1% penicillin–streptomycin in a humidified atmosphere at 37 °C with 5% CO_2_.

### 4.9. UVB Exposure In Vitro

Radiation from the UVB lamps (220 V, 20 W) (Beijing Lighting Research Institute, Beijing, China) ranged from 280 to 320 nm, with a peak at 312 nm. Cells were irradiated with UVB rays at an intensity of 25 μW/cm^2^, and the total dose was controlled by the irradiation time. In this study, cells were irradiated for 12 min to obtain a radiation exposure of 18 mJ/cm^2^. Cells were treated with RhFGF21 for 1 h before UVB irradiation. To prevent possible UVB absorption by proteins, the culture media were removed and stored and replaced with fresh PBS. Following UVB irradiation, PBS was discarded and the stored culture media were added back to the cells. The cells were then harvested and/or analyzed 6 h post-UVB irradiation.

### 4.10. Cell Viability

HaCaT cells were seeded into 96-well plates at a density of 1 × 10^4^ cells/well and cultured for 24 h. Then the cells were subjected to the indicated treatments for 6 h. The cells were incubated with 200 µL of 0.5 mg/mL MTT solution at 37 °C for 3 h. The supernatant was discarded, and the formazan was dissolved in 150 µL of dimethyl sulfoxide (DMSO). The optical density (A value) was measured at 490 nm wavelength using a microplate reader (Bio Tek, Winooski, VT, USA). Cell viability was calculated using the following equation: cell viability (%) = 1 − (Asample − Asample)/(Acontrol − Ablank) × 100%.

### 4.11. MDC Staining

MDC, an autofluorescent compound, staining reflects autophagy activity and is extensively used in autophagy research [43]. In this study, cells were stained with 0.05 mM MDC at 37 °C for 30 min and then imaged with a fluorescence microscope (Olympus, Tokyo, Japan).

### 4.12. Hoechst 33258 Staining

HaCaT cells were cultured in 6-well plates for 24 h and then subjected to the indicated treatments for 6 h. The cells were incubated with Hoechst 33258 at 37 °C for 30 min and then imaged with a fluorescence microscope (Olympus, Tokyo, Japan).

### 4.13. Reverse Transcription Polymerase Chain Reaction (RT-PCR)

TRIzol reagent (Invitrogen, Carlsbad, CA, USA) was used to extract total RNA samples from skin tissues. To synthesize cDNA, 2 μg of total RNA was reverse transcribed using the PrimeScriptTM RT reagent kit with gDNA Eraser (Perfect Real Time) (TaKaRa, Otsu, Japan). PCR was performed using a SYBR green-based RT-PCR kit (TaKaRa, Otsu, Japan) on a CFX Connect Real Time PCR detection system (Bio-Rad, Hercules, CA, USA). The primers (GenePharma, Suzhou, China) used for RT-PCR are listed in Table 1. After an initial denaturation step at 95 °C for 30 s, 40 cycles of PCR were carried out. Each cycle consisted of a melting step at 95 °C for 15 s and an annealing extension step at 60 °C for 40 s. The data were quantified by the 2-ΔΔCt method and normalized to ACTB expression.

### 4.14. Western Blot Analysis

Samples were lysed on ice in RIPA lysis buffer (Beyotime Institute of Biotechnology, Nanjing, China) for 1 h and then centrifuged at 12,000× *g* at 4 °C for 10 min. The supernatant was obtained and stored at −20 °C. Protein concentrations were determined with a BCA protein assay kit (Beyotime Institute of Biotechnology, Nanjing, China). Samples were added in 5× loading buffer and boiled for 10 min for denaturation. Fifty micrograms of the total protein in each sample was separated on a 10% SDS-polyacrylamide gel and then transferred to polyvinylidene fluoride (PVDF) membranes. The membranes were blocked with 5% non-fat milk in TBST at room temperature for 2 h and then incubated overnight with the corresponding primary antibodies at 4 °C. The membranes were washed three times with TBST, and then incubated with horseradish peroxidase-conjugated secondary antibodies at room temperature for 2 h. Protein blots were detected using enhanced chemiluminescence (ECL) reagents (Biological Industries, Beit HaEmek, Israel).

### 4.15. Immunofluorescence Staining

The cells were washed three times with PBS and fixed with 4% paraformaldehyde at 37 °C for 30 min, washed three times with PBS, permeabilized by the treatment with 0.1% Triton X-100 for 10 min, washed with PBS and blocked with 10% fetal bovine serum for 30 min. The cells were then incubated overnight with antibodies at 4 °C. Then, the coverslips were washed with PBS and incubated with fluorochrome-conjugated secondary antibodies at room temperature for 2 h. Afterward, the cells were washed three times with PBS to remove free antibodies and stained with DAPI for 10 min. Images were photographed with a fluorescence microscope (Nikon, Tokyo, Japan).

### 4.16. SiRNA Transfection

Human siRNA targeting AMPK (si-AMPK) and the negative control (si-con) were purchased from GenePharma (Shanghai, China). The sequences of the si-AMPK duplex were as follows: sense strand, 5’-GGACAGGGAAGCCUUAAAUTT-3’, and antisense strand, 5’-AUUUAAGGCUUCCCUGUCCTT-3’. The sequences of the negative control siRNA duplex were as follows: sense strand, 5’-UUC UCCGAACGUGUCACGUTT-3’, and antisense strand, 5’-ACGUGACACGUUCGGAGAATT-3’. Cells were transfected with 20 nM si-AMPK or si-con using Lipofectamine^®^ 2000 (Invitrogen, Carlsbad, CA, USA) according to the manufacturer′s instructions. The transfected cells were maintained for 48 h before use in experiments.

### 4.17. Statistical Analysis

All data were analyzed by SPSS17.0 system software. Means were compared among different groups by one-way analysis of variance (ANOVA) followed by the least significant difference (LSD) post-hoc test. All data are presented as the means ± SD. *p* < 0.05 was considered significant.

## Figures and Tables

**Table 1:** List of primer sequences used for RT-PCR in this study.

Gene	Primer Sequences
ACTB	5′-GTGCTATGTTGCTCTAGACTTCG-3′ 5′-ATGCCACAGGATTCCATACC-3′
Il1b	5′-AAGCCTCGTGCTGTCGGACC-3′ 5′-TGAGGCCCAAGGCCACAGGT-3′
Tnf	5′-CAAGGGACAAGGCTGCCCCG-3′ 5′-GCAGGGGCTCTTGACGGCAG-3′
IL6	5′-AGAAGGAGTGGCTAAGGACCAA-3′ 5′-AACGCACTAGGTTTGCCGAGTA-3′
COX2	5′-CCCTTGGGTGTCAAAGGTAA-3′ 5′-GCCCTCGCTTATGATCTGTC-3′

## Data Availability

Not applicable.
